# KCNH6 Enhanced Hepatic Glucose Metabolism through Mitochondrial Ca^2+^ Regulation and Oxidative Stress Inhibition

**DOI:** 10.1155/2022/3739556

**Published:** 2022-09-30

**Authors:** Ying-Chao Zhang, Feng-Ran Xiong, Cheng Cheng, Han Shen, Ru-Xuan Zhao, Juan-Juan Zhu, Lin Zhang, Jing Lu, Jin-Kui Yang

**Affiliations:** ^1^Beijing Key Laboratory of Diabetes Research and Care, Beijing Diabetes Institute, Beijing Tongren Hospital, Capital Medical University, Beijing 100730, China; ^2^Beijing Sijiqing Hospital, Beijing 100097, China

## Abstract

KCNH6 has been proven to affect glucose metabolism and insulin secretion both in humans and mice. Further study revealed that Kcnh6 knockout (KO) mice showed impaired glucose tolerance. However, the precise function of KCNH6 in the liver remains unknown. Mitochondria have been suggested to maintain intracellular Ca^2+^ homeostasis; ROS generation and defective mitochondria can cause glucose metabolism disorders, including type 2 diabetes (T2D). Here, we found that Kcnh6 attenuated glucose metabolism disorders by decreasing PEPCK and G6pase abundance and induced Glut2 and IRS2 expression. Overexpression of Kcnh6 increased hepatic glucose uptake and glycogen synthesis. Kcnh6 attenuated intracellular and mitochondrial calcium levels in primary hepatocytes and reduced intracellular ROS and mitochondrial superoxide production. Kcnh6 suppressed oxidative stress by inhibiting mitochondrial pathway activation and NADPH oxidase expression. Experiments demonstrated that Kcnh6 expression improved hepatic glucose metabolism disorder through the c-Jun N-terminal kinase and p38^MAPK^ signaling pathways. These results were confirmed by experiments evaluating the extent to which forced Kcnh6 expression rescued metabolic disorder in KO mice. In conclusion, KCNH6 enhanced hepatic glucose metabolism by regulating mitochondrial Ca^2+^ levels and inhibiting oxidative stress. As liver glucose metabolism is key to T2D, understanding KCNH6 functions may provide new insights into the causes of diabetes.

## 1. Introduction

KCN, a human ether-a-go-go-related gene, encodes KCN ion channel in the Kv11 family, which ranks 11th in abundance among different ion channel families [1]. The KCN potassium channel family includes three subfamilies: KCN1, comprising the KCNH2, KCNH6, and KCNH7 genes; KCN2, comprising the Kv11.1 and Kv11.2 genes; and KCN3, comprising the Kv11.3 gene [2]. In the KCN1 subfamily, the KCNH2 gene is mainly distributed in the myocardium, endocrine cells, central nervous system, and lymphocytes [3–6]. The KCNH6 gene is highly expressed in islets and the central nervous system, while the KCNH7 gene is predominantly expressed in dorsal root ganglia and islets [2, 7–10]. Studies have shown that KCN channels play crucial roles in insulin secretion [11]. Our group found that adult patients or mice with dysfunctional KCNH6 gene expression presented with hypoinsulinemia or hyperglycemia [12, 13]. Another study revealed that KCNH6 may cause endoplasmic reticulum (ER) stress in the liver and cell apoptosis [14].

Pancreatic hormones, including insulin and glucagon, may regulate glucose metabolism in the liver [15]. Glucose metabolism disorders are results of a liver unable to regulate the action of insulin, which can lead to type 2 diabetes (T2D) [16]. Oxidative stress is a cellular status when too many reactive oxygen species (ROS) are generated upon various stimulus and thus decrease antioxidant capacity. Under the oxidative stress, normal oxidation/antioxidation dynamic balance in the body is disrupted. Additionally, biological macromolecules, such as nucleic acids, proteins, and lipids, are impaired under oxidative stress, causing a serious state that disrupts normal life activities. ROS constitute a group of chemically active compounds with oxygen-containing functional groups; these compounds include hydrogen peroxide (H_2_O_2_), the superoxide anion (O_2_^−^), and nitric oxide (NO) [17, 18]. Under normal conditions, free radicals are continuously generated and removed to maintain a dynamic cellular balance. However, disruptive factors such as ischemia, hypoxia, and hyperglycemia cause multiple types of free radicals to be excessively produced in the body. In this case, when the antioxidant protection mechanism is insufficient, oxidative stress increases, leading to damage to body tissues and cells.

The sources of ROS include enzymatic, nonenzymatic, and mitochondrial pathways, with the latter considered the main ROS source [19]. Mitochondria are involved in several vital cellular processes, including intracellular Ca^2+^ homeostasis, ROS generation, energy metabolism, and initiation of apoptosis [20]. Ca^2+^ is a crucial secondary messenger in regulating cellular physiological functions in the body. Ca^2+^ overload damages mitochondrial function, which is the main reason for ROS generation in mitochondria [21].

Our previous study demonstrated the function of KCNH6 in insulin secretion, and Kcnh6 knockout mice showed impaired glucose tolerance [12]. Here, we want to examine the role of KCNH6 in the liver and identify the underlying molecular mechanism of this action. Adult Kcnh6 knockout (KO) mice showed glucose metabolism disorders, as indicated by significantly elevated oxidative stress and mitochondrial calcium levels. The results indicated that KCNH6 expression attenuated dysregulated liver glucose metabolism by regulating mitochondrial Ca^2+^ levels and preventing oxidative stress.

## 2. Materials and Methods

### 2.1. Animals

Wild-type and Kcnh6-null mice with C57BL/6 background were used in experiments. All animal experiments complied with the guidelines of the Ethics Review Committee at the Institute of Zoology, Capital Medical University, China (No. TRECKY2018-037).

### 2.2. Metabolic Assays and Measurements

Glucose tolerance test (GTT) and insulin tolerance test (ITT) were used as before [22]. The mice used in these tests were allowed free access to water. An automatic glucometer from Accu-Chek Performa (Roche, Basel, Switzerland) was used for glucose measurements. An ELISA kit (Millipore, MA, USA) was used to measure the insulin concentration.

### 2.3. Cell Culture and Treatment

Hepatocytes were maintained in DMEM (Corning, Invitrogen, MA, USA) with 4.5 g/L glucose and 10% fetal bovine serum (FBS; HyClone). HepG_2_ cells bought from Cell Resource Center (Beijing, China) were seeded in MEM (HyClone) containing 10% FBS. Cells were cultured within 30 h of plating at 37°C in humidified air with 5% CO_2_.

### 2.4. Cellular Glycogen Content Measurement

A glycogen assay kit (BioVision) was used to measure glycogen levels following the manufacturer's protocol [23].

### 2.5. Plasmids

The human KCNH6 gene cloned into a pcDNA3.1 vector (Sino-GenoMax, Shanghai, China) was used. TransIntro (Transgen, Beijing, China) was used to transfect HepG_2_ cells with plasmid DNA following the manufacturer's protocol.

Lentivirus carrying Kcnh6 (LV-Kcnh6, GeneChem, Beijing, China) was injected into male KO mice at age of 12 weeks via the tail vein (7 × 10^4^ TU/g body weight). Green fluorescent protein carried by lentivirus (GFP, LV-Ctrl) was used as a negative control. Subsequent experiments including GTT and western blot were all performed 2 weeks after lentivirus injection. GTT was performed after the mice fasted overnight for 16 hours. Blood glucose concentration was tested at 0, 15, 30, 60, and 120 min after glucose (2 g/kg body weight) injection with an automatic glucometer ACCU-CHEK Performa (Roche).

### 2.6. Glucose Uptake Measurement

2-[*N*-(7-Nitrobenz-2-oxa-1,3-diazol-4-yl) amino]-2-deoxy-D-glucose (2-NBDG, Life Technologies) was purchased for glucose uptake measurements [24]. Glucose uptake was detected by flow cytometry under basal conditions and after insulin stimulation. HepG2 cells were placed in 12-well plates after 24 h of preincubation. Then, cells were divided into three groups. Cells transfected with pcDNA3.1 were used as the control (Ctrl) group. The cells transfected with Kcnh6 were used as an experimental group (Kcnh6). The cells transfected with Kcnh6 and with E4031, an inhibitor of Kcnh6, composed another experimental group (Kcnh6+E4031). WT cells cultured without fluorescent 2-NBDG were used for gating. Fluorescent 2-NBDG was used to detect the Ctrl, Kcnh6, and Kcnh6+E4031 groups.

The cells were transferred to fresh culture with or without fluorescent 2-NBDG for 1 h. 2-NBDG uptake experiment was terminated once the medium was removed. The cells were then placed in 200 *μ*L of precooled fresh medium and cultured at 4°C for later flow cytometry analysis. 10,000 single cells were collected with a BD Accuri C6 flow cytometer (BD, NJ, USA) within 30 s. The FL1 fluorescence intensity was calculated after normalization to the WT signal.

### 2.7. Cytosolic and Mitochondrial Ca^2+^ Measurements

The cytosolic Ca^2+^ concentration ([Ca^2+^]_c_) in primary hepatocytes was determined with a confocal laser scanning microscope and 5 *μ*M calcium orange (MA, USA, Invitrogen). Calcium orange was dissolved in Hanks buffer with additional Pluronic F-127 (0.005%, Molecular Probes, MA, USA) at 37°C for 30 min following washing with Hanks buffer twice.

To measure the mitochondrial Ca^2+^ concentration ([Ca^2+^]_m_), the cells were seeded into a 15 mm cover glass-bottomed dish (NEST, Jiangsu, China) loaded with 4 *μ*M Rhod-2/AM (Invitrogen) at 37°C and incubated for 30 min. Then, the cells were cultured for another 30 min at 37°C before analyzing on a confocal microscope (FV1000, Olympus, Japan) with an inverted microscope at 60x oil immersion objective.

### 2.8. Mitochondrial Superoxide Production and Cellular ROS Measurements

ROS production in HepG2 cells or H_2_O_2_-treated HepG2 cells was detected by flow cytometry. The cells without plasmid transfection constituted the WT group. The cells transfected with pcDNA3.1 constituted the Ctrl group. The cells transfected with Kcnh6 constituted an experimental group (Kcnh6). The cells transfected with human Kcnh6 and with E4031 constituted another experimental group (Kcnh6+E4031). WT cells without dichlorodihydrofluorescein-diacetate (DCF-DA) were used for gating. DCF–DA was used with the Ctrl, Kcnh6, and Kcnh6+E4031 groups.

The cells were loaded with H_2_O_2_ (250 *μ*M) and with or without E4031 and maintained for 24 h. They were then incubated with 10 mM DCF-DA (Sigma, Germany) for 30 min at 37°C. They were collected after one wash with PBS. A total of 10,000 cells were harvested with Accuri C6 flow cytometer (BD, MA, USA) within 30 s. A fluorescence-activated cell sorting (FACS) flow cytometer was performed to measure the change in fluorescence intensity. The FL1 fluorescence intensity was calculated after normalization to the WT signal.

Compound-embedded tissues were cut at optimized section before loaded with 10 *μ*M dihydroethidium (DHE, Sigma) for 15 min. Fluorescence microscopy was used to analyze the sections.

### 2.9. Quantitative Real-Time Polymerase Chain Reaction (qRT–PCR)

RNA was isolated from HepG_2_ cells or liver. RNAprep Pure Tissue Kit or Cell/Bacteria Kit (Tiangen, Beijing, China) was used to quantify mRNA.

TransScript cDNA Synthesis SuperMix (Transgen) was used for cDNA synthesis. TransStart Top Green qPCR SuperMix (Transgen) was used to perform qRT–PCR. The *ΔΔ*Cq method was used for relative gene expression measurement. The primers used in the human and mouse experiments are listed in Supplementary Tables 1 and 2.

### 2.10. Western Blotting

Western blotting was used as before [12]. Antibodies are showed in Supplementary Table 3.

### 2.11. Data Analysis

Dara presented as the mean ± SEM were analyzed with GraphPad Prism software (Version 8.0). Statistical comparisons were calculated by unpaired-sample *t*-test or by Mann–Whitney *U* test for experiments with *n* < 6 samples. Significance was considered to be a *P* value < 0.05.

## 3. Results

### 3.1. KCNH6 Regulated Hepatic Glucose Metabolism in Kcnh6 Knockout Mice

**Figure 1:**
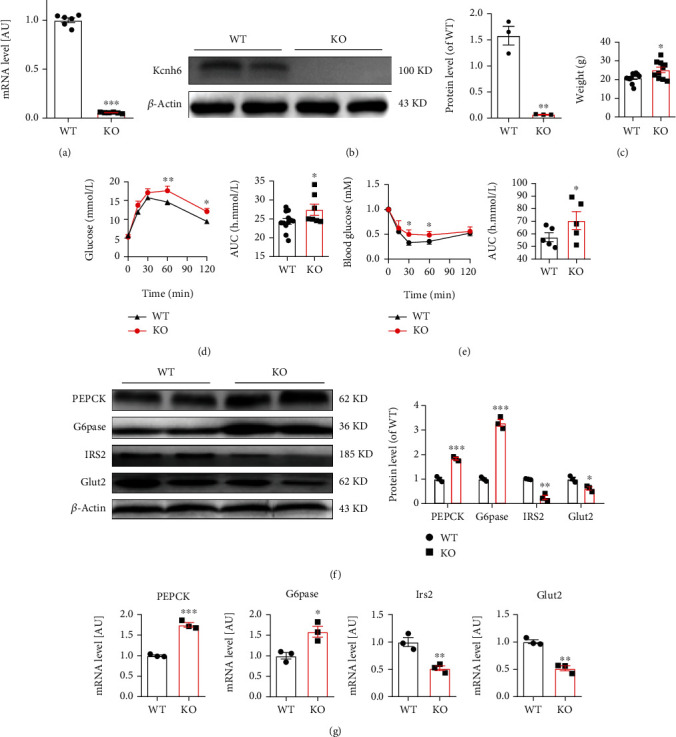
KCNH6 regulated hepatic glucose metabolism in Kcnh6 knockout mice. (a) Detection of the mRNA expression level of *Kcnh6* in wild-type (WT) and Kcnh6 knockout (KO) mice using qRT-PCR (*n* = 9). (b) Western blot results showing protein levels (*n* = 3). Disruption of glucose metabolism in KO mice was assessed by evaluating (c) body weight (*n* = 10), (d) performing glucose tolerance test (*n* = 12 for the WT group; *n* = 8 for the KO group), and (e) evaluating insulin tolerance test in mice (*n* = 5). (f) Western blot results showing the expression of G6pase, PEPCK, IRS2, and Glut2 in mice (*n* = 2). (g) G6pase, PEPCK, IRS2, and Glut2 expression in mouse liver tissues as determined by qRT-PCR (*n* = 3). ^∗^*P* < 0.05, ^∗∗^*P* < 0.01, and ^∗∗∗^*P* < 0.001 vs. WT. Statistical comparisons were calculated using (a, c–e) Mann–Whitney *U* test and (b, f, g) one unpaired-sample *t*-test.

The role of KCNH6 in glucose metabolism was investigated using wild-type (WT) and global Kcnh6 knockout (KO) mice with normal chow (NC) for 18–20 weeks. First, the expression of KCNH6 was significantly decreased in the liver of the KO mice compared with that in the WT group (Figures 1(a) and 1(b)). Kcnh6 KO mice weighed more than the WT mice (Figure 1(c)). Both the GTT and ITT volumes indicated development of impaired glucose regulation (Figures 1(d) and 1(e)). Genes related with glucose metabolism were measured. The glucose transporter 2 (Glut2) and insulin receptor substrate 2 (IRS2) levels decreased, while the phosphoenolpyruvate carboxykinase (PEPCK) and glucose-6-phosphatase (G6Pase) levels increased in the KO mice compared with the WT mice (Figures 1(f) and 1(g)). Our results indicated that KCNH6 expression attenuated glucose metabolism disorders by decreasing PEPCK and G6pase expression and inducing Glut2 and IRS2 expression.

### 3.2. KCNH6 Affected Liver Glucose Metabolism in HepG2 Cells

**Figure 2:**
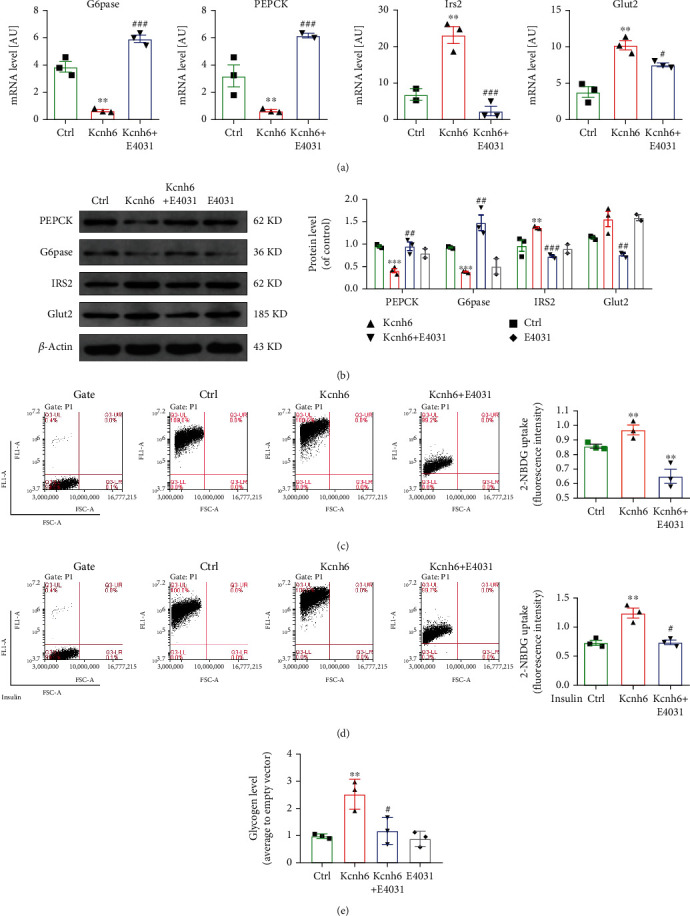
KCNH6 affected liver glucose metabolism in HepG2 cells. (a) Measurement of the G6pase, PEPCK, IRS2, and Glut2 levels in HepG2 cells expressing Kcnh6 as determined by qRT-PCR (*n* = 3). (b) Western blot results showing the G6pase, PEPCK, IRS2, and Glut2 levels in HepG2 cells overexpressing *KCNH6* (*n* = 3). Glucose uptake measurements were performed with flow cytometry at (c) the basal level (*n* = 3) and (d) after insulin stimulation (*n* = 3) in HepG2 cells overexpressing KCNH6. (e) Study of glycogen synthesis in HepG2 cells overexpressing *KCNH6* using a glycogen synthesis assay kit (*n* = 3). ^∗^*P* < 0.05, ^∗∗^*P* < 0.01, and ^∗∗∗^*P* < 0.001 vs. the Ctrl group. ^#^*P* < 0.05, ^##^*P* < 0.01, and ^###^*P* < 0.001 vs. the Kcnh6 group. Statistical comparisons were calculated using the Mann–Whitney *U* test.

Proteins related to glucose metabolism were measured both *in vivo* and *in vitro*. The overexpression of Kcnh6 in the HepG2 cells was verified at the protein and mRNA levels (Supplementary Figures 1A and 1B). The Glut2 and IRS2 levels both increased in *KCNH6*-overexpressing HepG_2_ cells, whereas the both the PEPCK and G6Pase levels decreased. After treatment with E4031, the expression of Glut2 and IRS2 decreased, while G6Pase and PEPCK increased (Figures 2(a) and 2(b)).

Next, the function of KCNH6 in glucose uptake and glycogen synthesis was investigated by flow cytometry. An apparent increase in both basal and insulin-stimulated glucose uptake was observed in Kcnh6-overexpressing HepG_2_ cell line. Further, this increase was reduced by treatment with E-4031, an inhibitor of hERG activity (Figures 2(c) and 2(d)). Similarly, glycogen synthesis increased in Kcnh6-overexpressing HepG_2_ also compared with the control cells, and these increases were reduced by E-4031 treatment (Figure 2(e)). Therefore, the overexpression of KCNH6 increased the glucose uptake and glycogen synthesis rates, confirming that KCNH6 affects liver glucose metabolism.

### 3.3. KCNH6 Attenuated Intracellular and Mitochondrial Calcium Levels in Primary Hepatocytes

**Figure 3:**
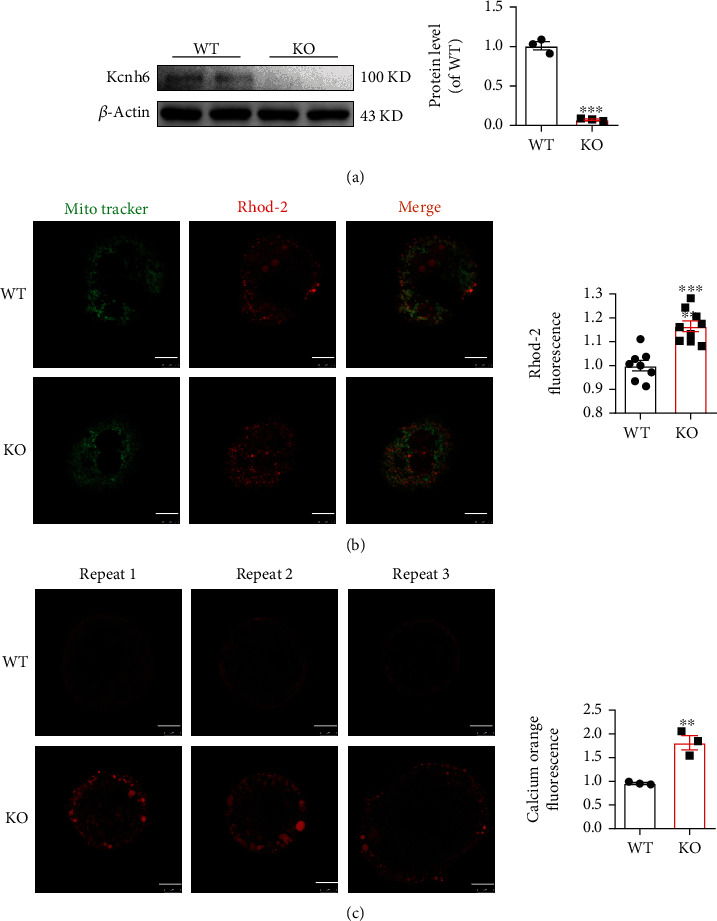
KCNH6 attenuated intracellular and mitochondrial calcium levels in primary hepatocytes. (a) Detection of *KCNH6* expression in the mitochondria (*n* = 3). (b) Calcium ion levels in mouse liver mitochondria as determined by Rhod-2-AM staining (*n* = 8 for the WT group; *n* = 10 for the KO group). (c) Fluo-4 AM calcium orange staining was performed for detecting changes in calcium ion levels in the cytoplasm of primary liver cells in mice (*n* = 3 for the WT group and the KO group). ^∗∗^*P* < 0.01 and ^∗∗∗^*P* < 0.001 vs. the WT group. Statistical comparisons were calculated using the (a, c) Mann–Whitney *U* test and (b) unpaired-sample *t*-test.

Our previous report revealed that KCNH6 knockout in pancreatic *β* cells caused increased calcium influx in the intermediate term, and an increase to an ultrahigh intracellular calcium concentration caused ER stress in the long term [13]. Notably, intracellular Ca^2+^ homeostasis is important for mitochondrial function, and Ca^2+^ overload exerts toxic effects on mitochondria, leading to ROS generation [21]. Therefore, whether KCNH6-mediated mitochondrial Ca^2+^ affects glucose metabolism disorders by driving ROS generation was explored. The expression of KCNH6 in hepatic mitochondria of WT mice was confirmed. We found that Kcnh6 was significantly downregulated in the KO mice (Figure 3(a)). The basal [Ca^2+^]_m_ in primary hepatocytes of KO mice was significantly increased compared with the hepatocytes in control mice (Figure 3(b)). Subsequently, calcium orange staining was performed to measure cytosolic Ca^2+^ in cells and thus determine the effects of Kcnh6 on cytosolic calcium levels. The results showed that the [Ca^2+^]_c_ in primary hepatocytes with *Kcnh6* knocked down was increased compared with that in control hepatocytes (Figure 3(c)). All the above-mentioned findings indicated that KCNH6 played a critical role in regulating mitochondrial Ca^2+^ concentrations in primary hepatocytes.

### 3.4. KCNH6 Reduced Intracellular ROS and Mitochondrial Superoxide Production

**Figure 4:**
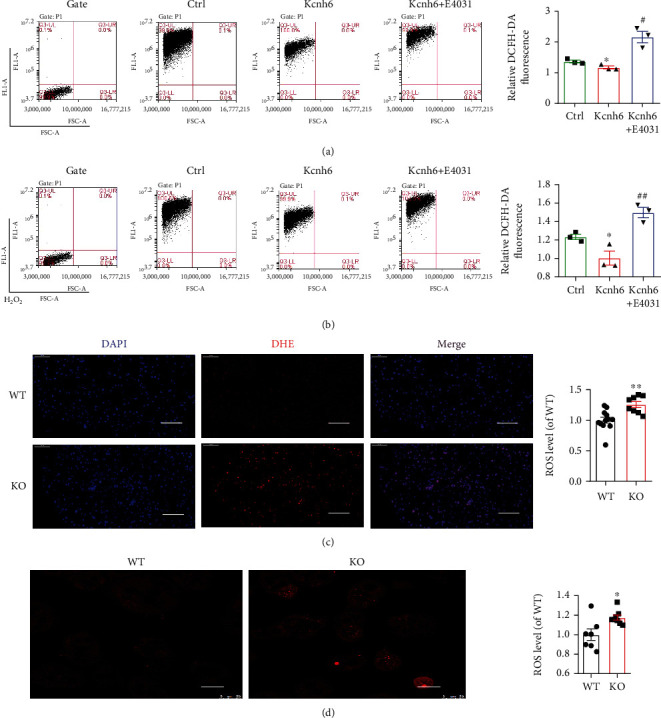
KCNH6 reduced intracellular ROS and mitochondrial superoxide production. ROS production in (a) HepG2 cells or (b) H_2_O_2_-treated HepG2 cells as detected by flow cytometry (*n* = 3). (c) ROS in mouse livers were detected by DHE staining (*n* = 12 for the WT group; *n* = 8 for the KO group). (d) Detection of mitochondrial superoxide in WT and Kcnh6 KO mice (*n* = 7 for the WT group; *n* = 8 for the KO group). ^∗^*P* < 0.05 and ^∗∗^*P* < 0.01 vs. the Ctrl group. ^#^*P* < 0.05 and ^##^*P* < 0.01 vs. the Kcnh6 group. Statistical comparisons were calculated using the (a, b) Mann–Whitney *U* test and (c, d) unpaired-sample *t*-test.

Next, we used DCF-DA to measure the intracellular ROS levels. A decrease in intracellular ROS levels was found in the Kcnh6-overexpressing HepG_2_ cells. E4031, a specific inhibitor of hERG, countered this effect (Figure 4(a)). Specifically, ROS production induced by H_2_O_2_ was significantly downregulated by E4031 treatment in KCNH6-overexpressing HepG_2_ cells (Figure 4(b)). ROS contents in the liver of both WT and KO mice were measured by DCF staining. ROS generation was found to be dramatically upregulated in the KO mice (Figure 4(c)). The data demonstrated that KCNH6 expression led to decreased ROS generation in both cells and mice.

Oxidases and the mitochondrial electron transport chain are both main cellular generation of ROS. ROS generated from mitochondria constitute the largest percentage of total ROS [19]. Mitochondrial ROS (mROS) were measured by the mitochondria-targeted biosensor MitoSOX, and results similar to those obtained with DCF-DA were found (Figure 4(d)). Nicotinamide adenine dinucleotide phosphate (NADPH) oxidase levels in cells and KO mice were measured to further analyze the production of ROS. NOX family genes, including NOX1–5, are expressed in different tissues and cells [25]. The results indicated that the levels of total p47phox, p22phox, p67phox, and gp91phox were decreased in KCNH6-overexpressing cells. After treatment with E4031, however, the protein levels of total p47phox, p22phox, p67phox, and gp91phox were increased (Supplementary Figure 2A). Similar results were found with KO mice (Supplementary Figures 2B and 2C). These results suggested that KCNH6 might suppress oxidative stress by inhibiting mitochondrial pathway activity and NADPH oxidase expression.

### 3.5. KCNH6 Improved Glucose Metabolism through the JNK and p38^MAPK^ Pathways

**Figure 5:**
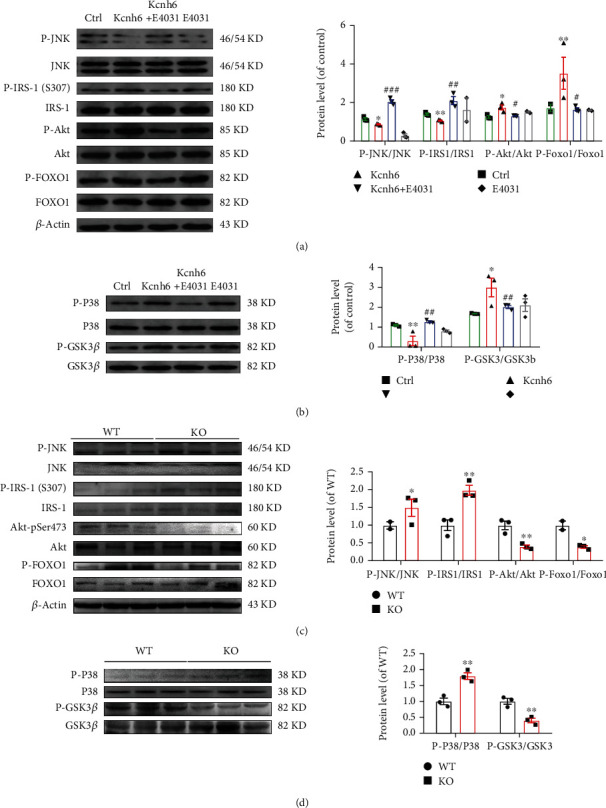
KCNH6 improved glucose metabolism through the JNK and p38^MAPK^ pathways. (a) Protein levels of JNK signaling pathway-related genes in HepG2 cells. (b) Protein levels of p38^MAPK^ signaling pathway-related genes in HepG2 cells. (c) Levels of JNK signaling pathway-related genes in WT and KO mice. (d) Protein expression levels of p38^MAPK^ signaling pathway-related genes in WT and KO mice. ^∗^*P* < 0.05 and ^∗∗^*P* < 0.01 vs. WT or Ctrl. ^#^*P* < 0.05 and ^##^*P* < 0.01 vs. Kcnh6 (*n* = 3, a–d). Statistical comparisons were calculated using the Mann–Whitney *U* test.

Excess ROS can impair cells in several ways, including oxidizing DNA, proteins, and lipids. Different stress-related intracellular pathways, including the NF-*κ*B, p38^MAPK^, and JNK/SAPK pathways, can be activated upon abundant ROS production, which ultimately results in impaired glucose metabolism [26, 27]. The results of this study showed that KCNH6-overexpressing cells exhibited decreased phosphatase and tensin homolog deleted on chromosome ten (PTEN) and phosphorylation of p38^MAPK^, JNK, and IRS-1, with increasing phosphorylation of Akt and GSK (Figures 5(a) and 5(b)). The same results were found in the KO mice. The KO mice showed upregulated phosphorylation of JNK and p38^MAPK^. An increase in the phosphorylation of IRS-1 and PTEN was also detected, accompanied by decreasing phosphorylation of Akt, FOXO1, and GSK (Figures 5(c) and 5(d)).

### 3.6. Forced KCNH6 Overexpression Ameliorated Hepatic Glucose Metabolism Disorders in *Kcnh6* KO Mice

**Figure 6:**
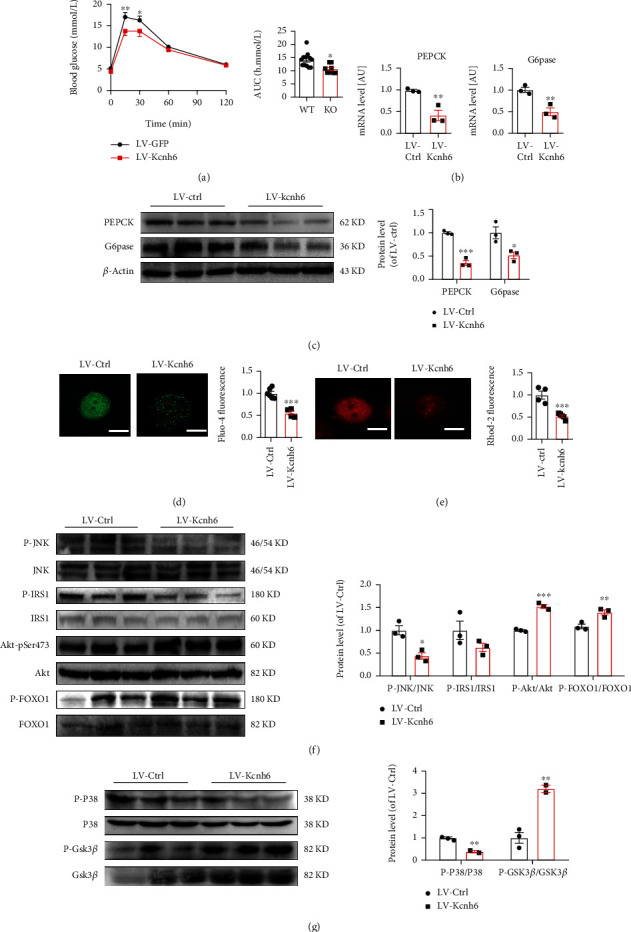
KCNH6 ameliorated hepatic glucose metabolism disorders in reversal KO mice. (a) Lentiviruses with GFP (LV-Ctrl) or Kcnh6 (LV-Kcnh6) were injected into 12-week-old male KO mice via the tail vein (7 × 10^4^ TU/g body weight). (a) Restoration of KCNH6 expression reversed the glucose metabolism disorder in KO mice (*n* = 14 for the WT group; *n* = 9 for the KO group). (b) mRNA levels of PEPCK and G6pase in the livers of mice detected by qRT-PCR (*n* = 3). (c) Levels of PEPCK and G6pase detected by western blotting (*n* = 3). (d) Fluo-4 AM staining for detecting changes in calcium ion levels in the cytoplasm of primary liver cells of KO mice (*n* = 6 for the WT group; *n* = 4 for the KO group). (e) Calcium ion levels of mitochondria in the KO mouse liver as detected by Rhod-2-AM staining (*n* = 4 for the WT group; *n* = 5 for the KO group). (f) Protein levels of the JNK signaling pathway as detected by western blotting (*n* = 3). (g) Western blotting was performed for measuring of the protein levels of genes related to the p38MAPK signaling pathway in the LV-Ctrl and LV-Kcnh6 mice (*n* = 3). ^∗^*P* < 0.05, ^∗∗^*P* < 0.01, and ^∗∗∗^*P* < 0.001 vs. the LV-Ctrl group; *n* = 6 mice in each group. Statistical comparisons were calculated using the (b–g) Mann–Whitney *U* test and (a) unpaired-sample *t*-test.

Hepatic glucose metabolism disorders in KO mice prompted us to investigate whether KCNH6 overexpression reverses glucose metabolism disorders. Therefore, lentivirus with GFP (LV-Ctrl) or Kcnh6 (LV-Kcnh6) was injected into the tail vein of KO mice to induce Kcnh6 overexpression (Supplementary Figures 3A and 3B). As expected, the restoration of Kcnh6 expression reversed the glucose metabolism disorder in the KO mice (Figure 6(a)). Specifically, LV-Kcnh6 reduced the expression of PEPCK and G6pase (Figures 6(b) and 6(c)). Furthermore, cytosolic and mitochondrial calcium levels were decreased in liver samples taken from the LV-Kcnh6-treated KO mice compared to their levels in control mice (Figures 6(d) and 6(e)). Additionally, LV-Kcnh6 decreased the phosphorylation of JNK and p38^MAPK^ (Figures 6(f) and 6(g)). In summary, the findings of this study support the hypothesis that glucose metabolism can be enhanced by increasing KCNH6 expression, which reduces mitochondrial calcium levels, ROS generation, and JNK and p38^MAPK^ signaling pathway activity.

## 4. Discussion

**Figure 7:**
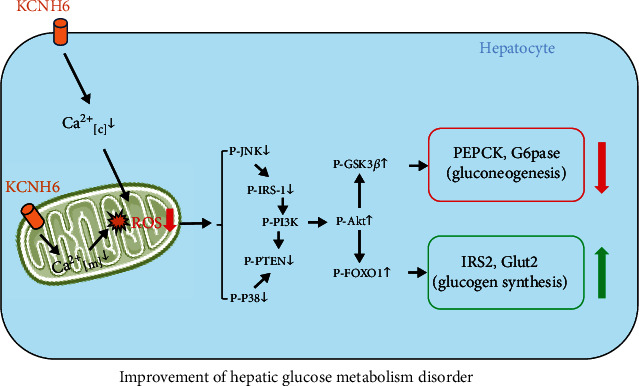
Cartoons depicting the function of KCNH6 in hepatic cells with oxidative stress and disordered glucose metabolism.

Glucose metabolism disorders precede the development of T2D, which may be induced in the liver and muscles in vivo. Oxidative stress is closely associated with the development of metabolic disorders [28]. Our previous studies revealed that Kcnh6 KO mice presented with impaired glucose tolerance or diabetes, which prompted us to explore the role played by KCNH6 in glucose metabolism [12]. Here, we want to detect the effect of KCNH6 on hepatic cells, which also play important roles in glucose metabolism. We used global Kcnh6 KO and KCNH6-overexpressing mice and found that KCNH6 reduced mitochondrial and intracellular ROS levels by reducing mitochondrial and intracellular calcium levels and ultimately protected against oxidative stress and glucose metabolism disorders mediated via the JNK and p38^MAPK^ signaling pathways (Figure 7).

Oxidative stress explains oxidant-antioxidant imbalance. Increasing evidence shows that sustained oxidative stress may cause T2D, which involves impaired insulin secretion and insulin resistance. For example, redox processes play important roles in lipid and carbohydrate metabolism, with H_2_O_2_ exerting an effect like insulin [29]. Another study found that ROS production enhanced insulin sensitivity [30]. Hyperglycemia associated with the formation of ROS, in particular, induced glucotoxicity [31]. Postprandial oxidative stress has been shown to cause metabolic disorders such as diabetes and obesity [32]. Here, we found that global Kcnh6 KO mice presented with liver glucose metabolism disorders. ROS production induced by H_2_O_2_ was decreased in Kcnh6-overexpressing HepG_2_ cells, and ROS production was dramatically increased in KO mice. NADPH oxidase expression was also found to be increased in the KO mice. Thus, our data strongly indicate that *KCNH6* might suppress oxidative stress by inhibiting mitochondrial pathway activity and NADPH oxidase expression.

Mitochondria play roles in metabolic disorders by modulating neuroendocrine, inflammatory, and transcriptional upon the acute psychological stress [33]. The mitochondrial redox signaling pathway is activated under the stimulus of hypoxia [34]. The concentration of calcium ions in mitochondria affects mitochondrial ATP synthesis, mitochondrial permeability transition *pore* (mPTP) opening, ROS production, cytoplasmic calcium signaling maintenance, and cytosolic calcium homeostasis. Therefore, maintaining normal mitochondrial calcium ion levels has important physiological implications, and abnormalities in these ion levels are associated with many important diseases. Previous studies have confirmed that the remodeling of mitochondrial Ca^2+^ homeostasis may be the main driver of ROS production. Several possible mechanisms have been suggested, including an increased metabolic rate induced by Ca^2+^ and induction of mPTP opening by Ca^2+ 28^. In this work, we identified that the expression of Kcnh6 was decreased in mitochondria. Then, we found that the basal [Ca^2+^]_m_ concentration of primary hepatocytes in KO mice was higher than the control hepatocytes. After rescue by LV-Kcnh6 infection, KO mice showed decreased cytosolic and mitochondrial calcium levels. Hence, KCNH6 might protect hepatocytes from oxidative stress by maintaining intracellular calcium homeostasis.

ROS are second messengers signaling molecules [35, 36]. They activate many redox-sensitive pathways including the nuclear factor kappa (NF-*κ*B) signaling pathways [37], the P38 mitogen-activated protein kinase (P38^MAPK^) pathway [38, 39], and the c-Jun N-terminal kinase (JNK) pathway [40]. The essential roles played JNK and p38^MAPK^ signaling in the initiation and progression of metabolic disorders have been well recognized. This study found that ROS generation was significantly increased in KO mice. Using *in vitro* and *in vivo* approaches, an inverse relationship was identified between KCNH6 expression and JNK and P38 activation when ROS levels were high. JNK and p38^MAPK^ can phosphorylate different targets, including IRS proteins and insulin receptors [36]. Phosphorylation of IRS-1 reduces its capacity for tyrosine phosphorylation and promotes IRS-1 degradation, accompanied by a decrease in Akt/protein kinase B phosphorylation [37]. Decreased phosphorylation of Akt can reduce the phosphorylation rate of FoxO1, leading to its inactivation in the nucleus, thereby increasing the transcriptional regulation of the key gluconeogenesis genes PEPCK and G6pase. Increased expression of G6Pase and PEPCK promotes hepatic gluconeogenesis and elevated blood glucose. GSK-3 is a serine protein kinase related to the glycogen regulation that phosphorylates and inactivates glycogen synthase. Decreased phosphorylation of Akt thus reduces P-GSK-3*β* levels, decreases glycogen synthase activity, inhibits cellular uptake of glucose and synthesis of glycogen, and increases blood glucose levels.

Although our study revealed that KCNH6 can prevent oxidative stress and maintain mitochondrial calcium homeostasis, the mechanism needs further investigation. In the future work, we may perform RNA-seq and other experiments to have a more comprehensive understanding of the downstream effects of KCNH6. Nevertheless, our study provides new insights into the increase in liver oxidative stress levels induced by mitochondrial calcium ion overload, which ultimately leads to disordered hepatic glucose metabolism, and into the therapeutic value of KCNH6*-*targeted drugs.

## Data Availability

All data generated or analyzed during this study are included in this published article (and its supplementary information files).

## Supplementary Materials

Supplementary 1 Supplementary Table 1: sequence of human primers for qRT-PCR. Supplementary Table 2: sequence of mouse primers for qRT-PCR. Supplementary Table 3: specific antibodies for western blotting.

Supplementary 2 Supplementary Figure 1: expression level of KCNH6. (A) Protein levels of Kcnh6 in HepG2 cells (*n* = 3). (B) mRNA level of *Kcnh6* in HepG2 cells (*n* = 3). ^∗∗^*P* < 0.01 and ^∗∗∗^*P* < 0.001 vs. the Ctrl group; statistical comparisons were calculated using the Mann–Whitney *U* test. Supplementary Figure 2: KCNH6 inhibited mitochondrial pathway activity and NADPH oxidase expression. Protein levels of p47phox, p22phox, p67phox, and gp91phox in HepG2 cells (A) and mice (B) (*n* = 3). (C) qRT-PCR results of the mRNA expression levels of different genes in mouse liver tissues (*n* = 3). ^∗^*P* < 0.05, ^∗∗^*P* < 0.01, and ^∗∗∗^*P* < 0.001 vs. the Ctrl group. ^#^*P* < 0.05, ^##^*P* < 0.01, and ^###^*P* < 0.001 vs. the Kcnh6 group. Statistical comparisons were calculated using the Mann–Whitney *U* test. Supplementary Figure 3: KCNH6 was overexpressed in Kcnh6 KO mice. Lentiviral particles with GFP (LV-Ctrl) or KCNH6 (LV-Kcnh6) were injected into 12-week-old male KO mice via the tail vein (7 × 10^4^ TU/g body weight). (A) Expression of LV-Ctrl and LV-Kcnh6 in mice was detected with a fluorescence microscope. (B) Protein levels of Kcnh6 in the livers of mice were measured by western blotting. ^∗∗^*P* < 0.01 vs. the LV-Ctrl group; *n* = 3 mice in each group. Statistical comparisons were calculated using the Mann–Whitney *U* test.
