# Supplementary material for: KCNH6 Enhanced Hepatic Glucose Metabolism through Mitochondrial Ca2+ Regulation and Oxidative Stress Inhibition
Source: Oxid Med Cell Longev. 2022 Sep 30;2022:3739556. doi: 10.1155/2022/3739556 (PMC9547380; doi:10.1155/2022/3739556)
Supplement: Supplementary 1 — Supplementary Table 1: sequence of human primers for qRT-PCR. Supplementary Table 2: sequence of mouse primers for qRT-PCR. Supplementary Table 3: specific antibodies for western blotting. [file 3739556.f1.docx]

Supplementary Table 1. Sequence of human Primers for qRT-PCR

| Gene | Forward (5’-3’) | Reverse (5’-3’) |
| --- | --- | --- |
| Kcnh6 | GTCGCTCCCCAAAACACTTA | CGAAGAGTTCGCAGAAGCC |
| G6pase | CATTGACACCACACCCTTTG | CCTGTACATGCTGGAGTTGA |
| IRS2 | GCAGAACATCCACGAGACCA | GGAACTCGAAGAGCTCCTTG |
| PEPCK | GCTACAACTTCGGCAAATAC | GGAAGATCTTGGGCAGTTTG |
| GLUT2 | AATTGCTCCAACCGCTCTCA | TAATAAGAATGCCCGTGACG |
| P22 phox | CTGGCAGTTCGCTGCTTACG | AGATCGCAGCGTGTAGAACC |
| P47 phox | GACCTCACCGAGAAGCTGAT | ACAAGGTGGTCTGCCTCGTT |
| P67 phox | AGTTCCCAATCCTTCGTCTGT | GAGCTCAAGGCGGAGCTAAA |
| P91 phox | GTGGTTCTACACCAGGGTCC | GGAAGGTGATGTTCCGATCC |
| β-actin | TCATGAAGTGTGACGTGGACATC | CAGGAGGAGCAATGATCTTGATCT |

Supplementary Table 2. Sequence of mouse Primers for qRT-PCR

| Gene | Forward (5’-3’) | Reverse (5’-3’) |
| --- | --- | --- |
| Kcnh6 | GCAATGTGTCACCCAACACC | ATGAACTCCTTGACCCGCAG |
| G6pase | CGACTCGCTATCTCCAAGTG | GGGCGTTGTCCAAACAGAAT |
| IRS2 | ACCGACTTGGTCAGCGAAG | CACGAGCCCGTAGTTGTCAT |
| PEPCK | CTGCATAACGGTCTGGACTT | GCCTTCCACGAACTTCCTCA |
| Glut2 | TCAGAAGACAAGATCACCGGA | GCTGGTGTGACTGTAAGTGGG |
| P22 phox | CTGGCAGTTCGCTGCTTACG | AGATCGCAGCGTGTAGAACC |
| P47 phox | GACCTCACCGAGAAGCTGAT | ACAAGGTGGTCTGCCTCGTT |
| P67 phox | AGTTCCCAATCCTTCGTCTGT | GAGCTCAAGGCGGAGCTAAA |
| P91 phox | GTGGTTCTACACCAGGGTCC | GGAAGGTGATGTTCCGATCC |
| β-actin | GTGACGTTGACATCCGTAAA | GCCGGACTCATCGTACTCC |

Supplementary Table 3. Specific Antibodies for Western blotting

| Gene | Company | Dilution | No. |
| --- | --- | --- | --- |
| Kcnh6 | Sigma-Aldrich | 1:1000 | SAB2104246 |
| IRS-1 | Cell signaling technology | 1:1000 | #2382 |
| phospho-IRS-1 (Ser307) | Cell signaling technology | 1:1000 | #2381 |
| p38MAPK | Cell signaling technology | 1:1000 | #8690 |
| phosphop38MAPK(Thr180/Tyr182) | Cell signaling technology | 1:1000 | #4511 |
| Akt | Cell signaling technology | 1:1000 | #9272 |
| phospho-Akt (Ser473) | Cell signaling technology | 1:1000 | #4060 |
| GSK | Cell signaling technology | 1:1000 | #12456 |
| phospho-GSK (Ser9) | Cell signaling technology | 1:1000 | #9323 |
| p-JNK | Cell signaling technology | 1:1000 | #4668 |
| JNK | Cell signaling technology | 1:1000 | #9252 |
| IRS2 | Cell signaling technology | 1:1000 | #3089 |
| FOXO1 | Cell signaling technology | 1:1000 | #2880 |
| phospho-FOXO1 (Ser256) | Cell signaling technology | 1:1000 | #84192 |
| β-actin | Cell signaling technology | 1:1000 | #8457 |
| PTEN | Cell signaling technology | 1:1000 | #9188 |
| phospho-PTEN(Ser380/Thr382/Thr383) | Cell signaling technology | 1:1000 | #9554 |
| PEPCK | abcam | 1:1000 | ab70359 |
| G6Pase | abcam | 1:1000 | ab83690 |
| Glut2 | abcam | 1:1000 | ab54460 |
| p47phox | abcam | 1:1000 | ab795 |
| p22phox | abcam | 1:1000 | ab75941 |
| p67phox | abcam | 1:1000 | ab175293 |
| p91phox | abcam | 1:1000 | ab80508 |
