# Supplementary figures and images for: KCNH6 Enhanced Hepatic Glucose Metabolism through Mitochondrial Ca2+ Regulation and Oxidative Stress Inhibition
Source: Oxid Med Cell Longev. 2022 Sep 30;2022:3739556. doi: 10.1155/2022/3739556 (PMC9547380; doi:10.1155/2022/3739556)

**A**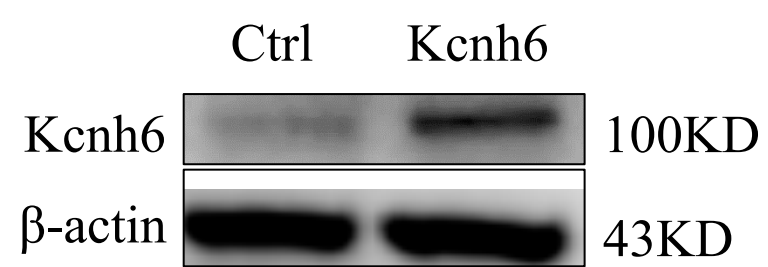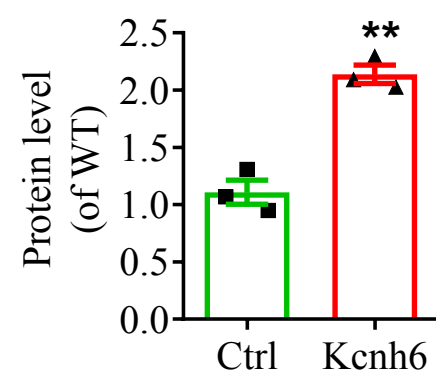**B**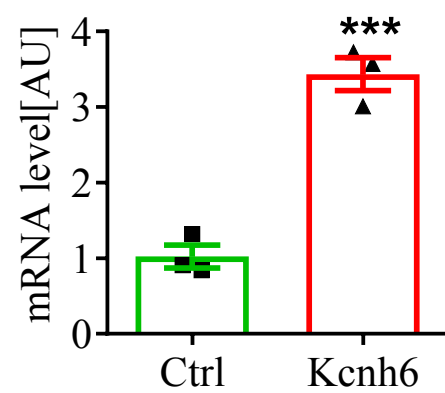

**A**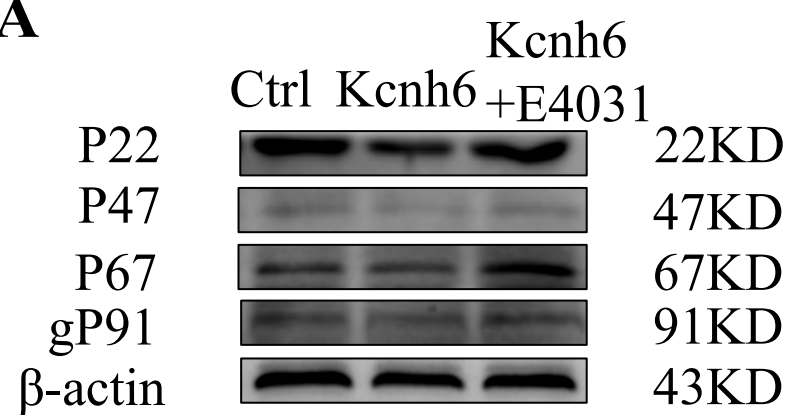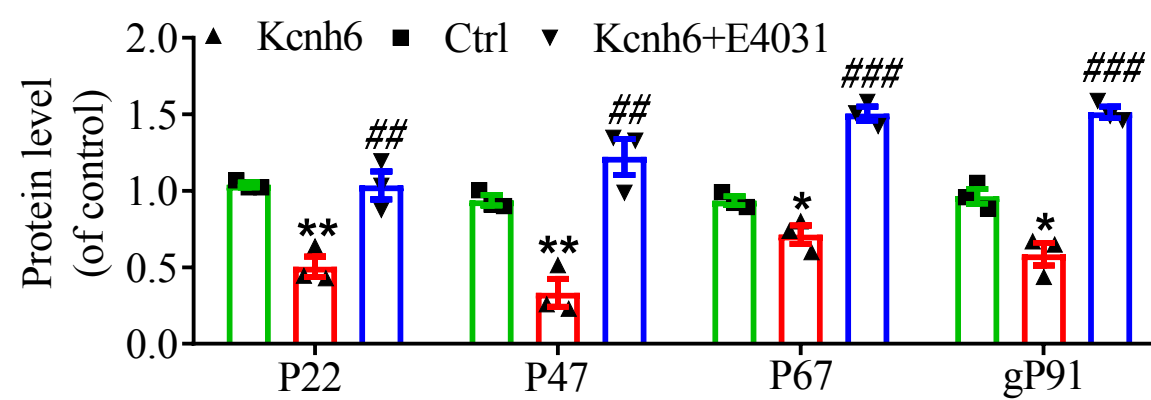**B**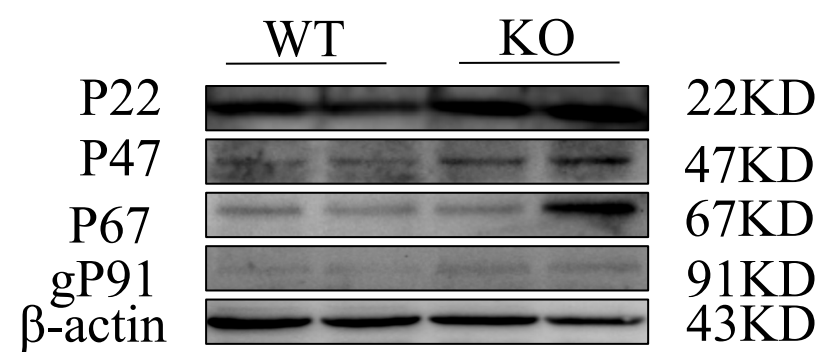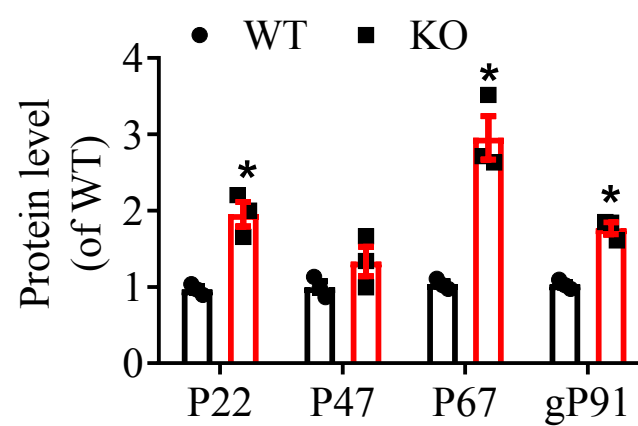**C**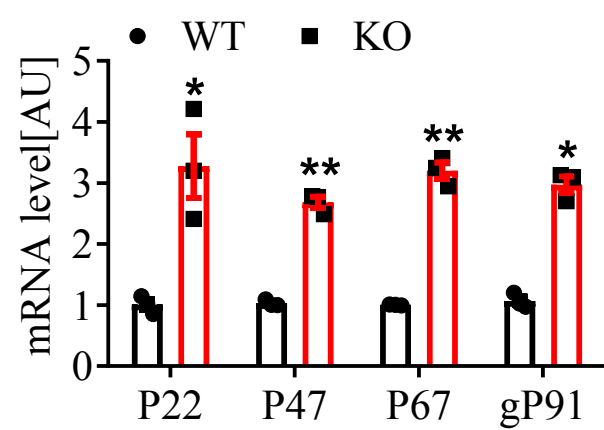

**A**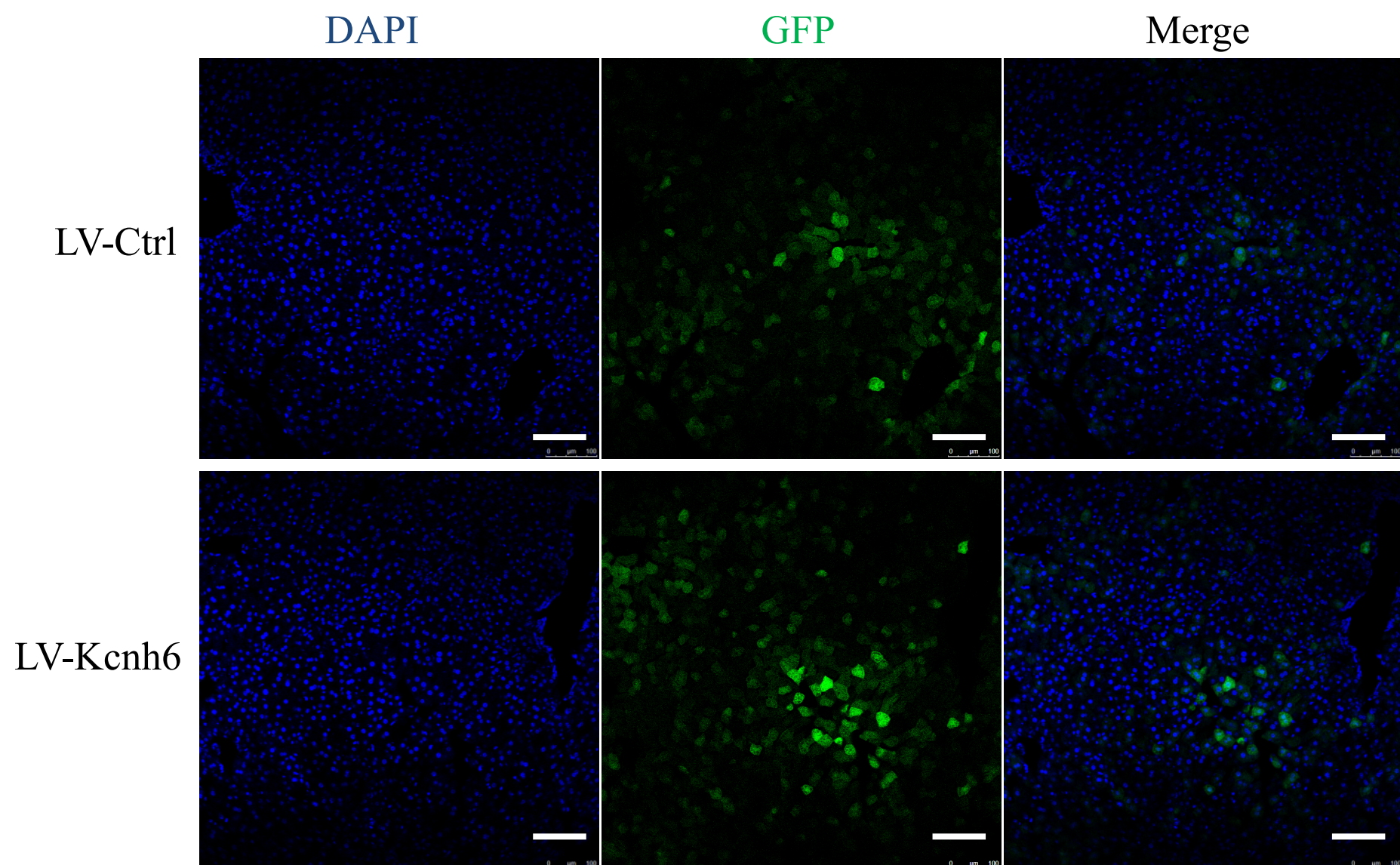**B**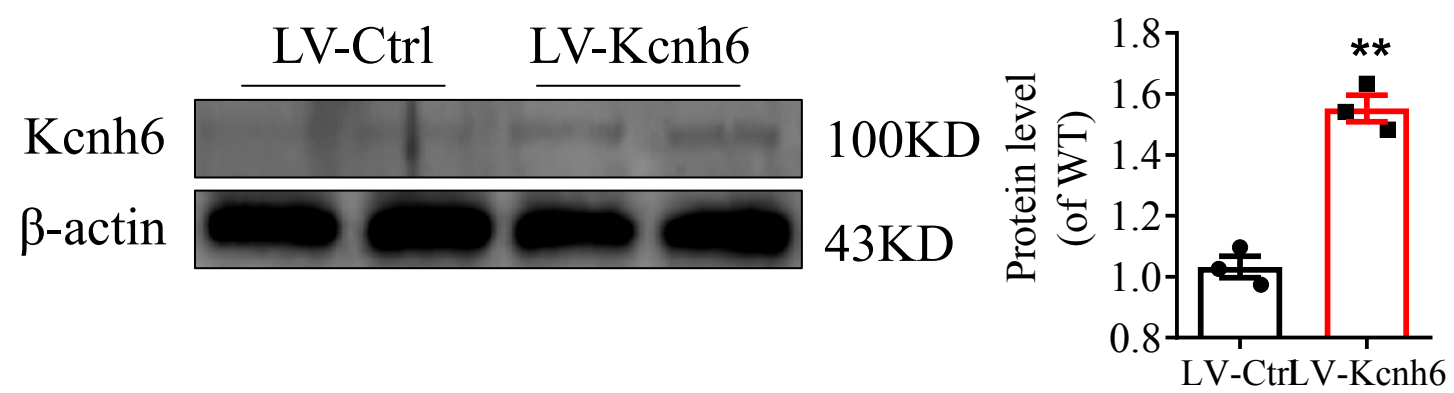

Supplement: Supplementary 2 — Supplementary Figure 1: expression level of KCNH6. (A) Protein levels of Kcnh6 in HepG2 cells (n = 3). (B) mRNA level of Kcnh6 in HepG2 cells (n = 3). ∗∗P < 0.01 and ∗∗∗P < 0.001 vs. the Ctrl group; statistical comparisons were calculated using the Mann–Whitney U test. Supplementary Figure 2: KCNH6 inhibited mitochondrial pathway activity and NADPH oxidase expression. Protein levels of p47phox, p22phox, p67phox, and gp91phox in HepG2 cells (A) and mice (B) (n = 3). (C) qRT-PCR results of the mRNA expression levels of different genes in mouse liver tissues (n = 3). ∗P < 0.05, ∗∗P < 0.01, and ∗∗∗P < 0.001 vs. the Ctrl group. #P < 0.05, ##P < 0.01, and ###P < 0.001 vs. the Kcnh6 group. Statistical comparisons were calculated using the Mann–Whitney U test. Supplementary Figure 3: KCNH6 was overexpressed in Kcnh6 KO mice. Lentiviral particles with GFP (LV-Ctrl) or KCNH6 (LV-Kcnh6) were injected into 12-week-old male KO mice via the tail vein (7 × 10^4^ TU/g body weight). (A) Expression of LV-Ctrl and LV-Kcnh6 in mice was detected with a fluorescence microscope. (B) Protein levels of Kcnh6 in the livers of mice were measured by western blotting. ∗∗P < 0.01 vs. the LV-Ctrl group; n = 3 mice in each group. Statistical comparisons were calculated using the Mann–Whitney U test. [file 3739556.f2.pdf]
